# Zn_0.4_Mg_0.6_Fe_2_O_4_ nanoenzyme: a novel chemo-sensitizer for the chemotherapy treatment of oral squamous cell carcinoma†

**DOI:** 10.1039/d2na00750a

**Published:** 2022-12-28

**Authors:** Liang Chen, Qingmei Kong, Mingxing Tian, Qian Zhang, Chengwan Xia, Chao Deng

**Affiliations:** a Department of Oral and Maxillofacial Surgery, Yi Ji Shan Hospital of Wannan Medical College Wuhu Anhui China; b Department of Oral and Maxillofacial Surgery, Nanjing Stomatology Hospital, Medical School of Nanjing University Nanjing China 2665927917@qq.com; c Key Laboratory of Non-coding RNA Transformation Research of Anhui Higher Education, School of Stomatology, Wannan Medical College Anhui China 20120015@wnmc.edu.cn

## Abstract

Hypoxic and acidic environments are the two main components of the microenvironment contributing to the poor efficacy of chemotherapy drugs in the treatment of oral squamous cell carcinoma (OSCC). In this study, we synthesized a series of Zn_1–*x*_Mg_*x*_Fe_2_O_4_ nanomaterials with enzyme-like properties, including catalase (CAT)-like, peroxidase (POD)-like, and glutathione (GSH)-like activity in an acidic environment. Among them, Zn_0.4_Mg_0.6_Fe_2_O_4_ performed the best and effectively increased the efficacy of doxorubicin (DOX) chemotherapy for the treatment of OSCC with reduced cardiotoxicity. Therefore, Zn_0.4_Mg_0.6_Fe_2_O_4_ could serve as a novel chemosensitizer in the treatment of OSCC.

## Abstract 2

Zn_0.4_Mg_0.6_Fe_2_O_4_ could serve as a promising novel chemo-sensitizer in the treatment of OSCC.

## Graphical abstract

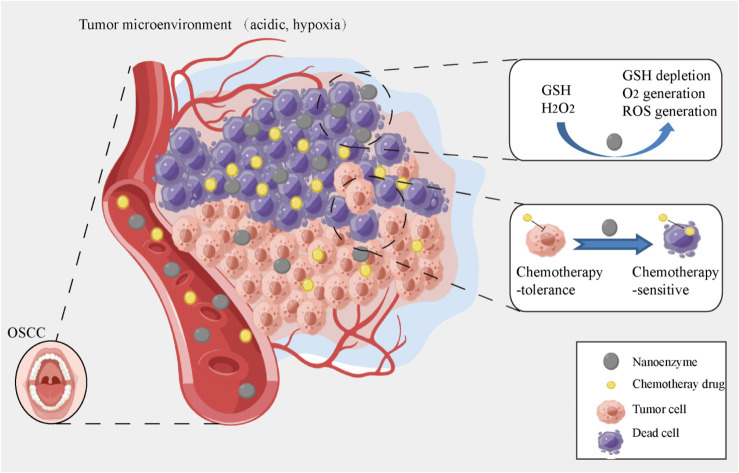

## 1 Introduction

As one of the most malignant tumor-prone sites, the number of new cases and associated deaths due to malignant tumors in the oral cavity worldwide were 377 713 and 177 757 respectively in 2020, and the numbers are increasing every year.^1^ Among these, oral squamous cell carcinoma (OSCC) accounts for 80–90%. Although radical resection is the preferred treatment, chemotherapy is still an important auxiliary method to treat or prolong life for patients with advanced/recurrence OSCC or patients who cannot tolerate surgery.^2^ The armamentarium of available chemotherapy has been increasing rapidly in recent years, while the 5-year survival rate of advanced/recurrence OSCC remains less than 30%.^3,4^ How to improve chemotherapy efficacy has become the key to prolonging the survival of patients with OSCC.

Hypoxic and acidic environments are two important characteristics of the tumor microenvironment. Oxygen is not only an important microenvironmental factor in the development of the organism and normal tissue homeostasis, but is also essential for oxidative metabolism, ATP production, and cell survival.^5^ In the process of tumor occurrence and development, the abnormal vascular system in the tumor microenvironment leads to obstacles in oxygen delivery, and at the same time, increased oxygen consumption due to tumor cell proliferation and immune cell infiltration eventually leads to hypoxia in the tumor microenvironment.^6^ Many studies have suggested that the hypoxic environment is closely related to tumor metastasis, tolerance to radiotherapy/chemotherapy, and a poor prognosis.^7,8^ Moreover, the lack of oxygen in tumor tissues also leads to an increase in anaerobic glycolysis and to the production/secretion of H^+^ in cells. Eventually, the acidic metabolites accumulate due to the limited blood perfusion in the acidic environment in malignant tumor tissues. Studies have shown that an acidic environment could activate proteolytic enzyme activity, which is involved in tumor tissue remodeling and tumor invasion.^9^ Furthermore, the degree of acidity of tumor tissues has also been reported to be related to their degree of malignancy.^10^ Therefore, this feature may be exploited as a novel approach to overcome chemotherapy tolerance in patients with OSCC by designing drugs to correct the hypoxic state based on the acidic tumor environment.

Since Yan *et al.* reported the intrinsic peroxidase-like activity of ferromagnetic nanoparticles in 2007,^11^ an increasing number of nanomaterials with enzyme-like characteristics (nanozymes) have been developed and applied in the biomedical field.^12^ Compared to traditional natural enzymes, nanozymes are generally of low-cost and can be mass-produced. Additionally, previous studies have shown that catalase (CAT)/peroxidase (POD) mimic nanozymes such as MnFe_2_O_4_ could catalyze H_2_O_2_ oxygen production, which could correct the hypoxic state in tumor tissue and may produce reactive oxygen species (ROS) that could inhibit tumor cell proliferation.^13–15^ This suggests that we may be able to correct the hypoxic state by constructing appropriate nanozymes that reduce tolerance to chemotherapy in OSCC patients.

**Fig. 1:**
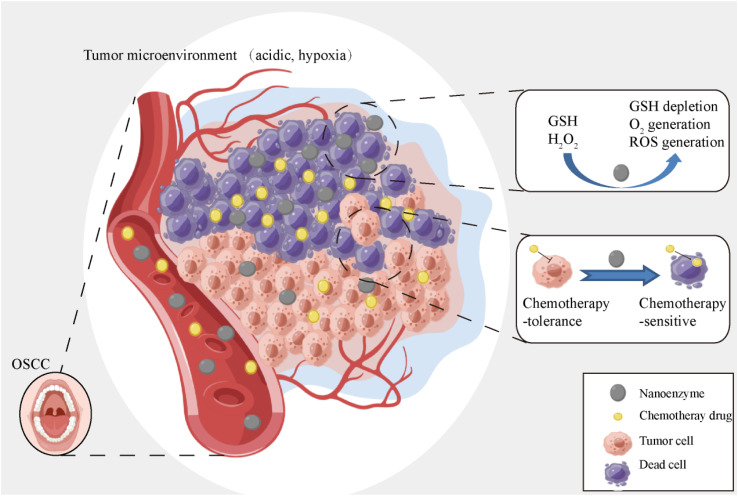
Diagram of the chemosensitization mechanism of the Zn_0.4_Mg_0.6_Fe_2_O_4_ nanoenzyme.

In this paper, a series of Zn_1–*x*_Mg_*x*_Fe_2_O_4_ nanozymes were synthesized and, of these, Zn_0.4_Mg_0.6_Fe_2_O_4_ exhibited the highest catalytic efficiency of POD-like, CAT-like, and GSH-like activity. Furthermore, the properties of POD–CAT and glutathione (GSH) activity of Zn_0.4_Mg_0.6_Fe_2_O_4_ were verified *in vitro*. Finally, we verified whether Zn_0.4_Mg_0.6_Fe_2_O_4_ could reduce chemotherapy tolerance and improve the effect of chemotherapy on OSCC, and the results showed that Zn_0.4_Mg_0.6_Fe_2_O_4_ could serve as a promising chemosensitizer in OSCC management. The main scheme of our study is depicted below (Fig. 1).

## 2 Results and discussion

### 2.1 Synthesis and characterization of Zn_1–*x*_Mg_*x*_Fe_2_O_4_

**Fig. 2:**
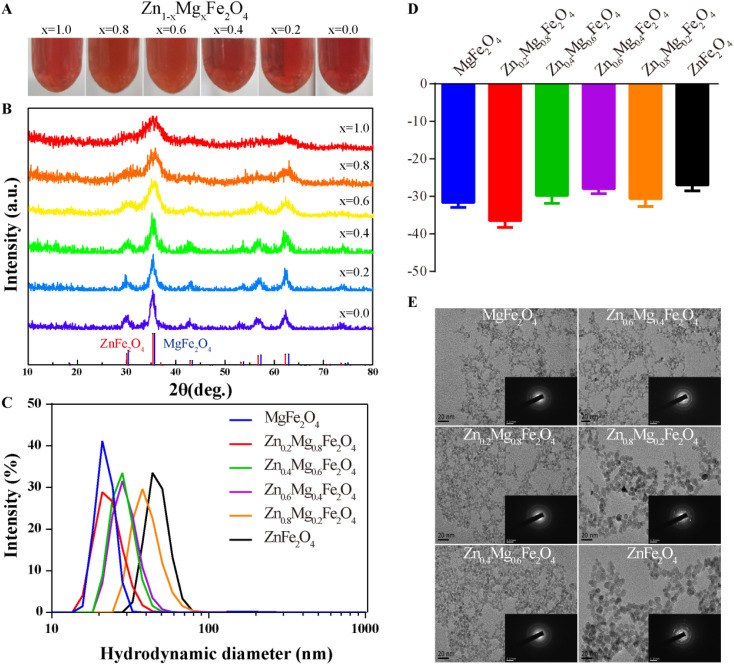
Characterization of Zn_1–*x*_Mg_*x*_Fe_2_O_4_. (A) Camera images of Zn_1–*x*_Mg_*x*_Fe_2_O_4_, (B) XRD spectra of Zn_1–*x*_Mg_*x*_Fe_2_O_4_, (C) DLS of Zn_1–*x*_Mg_*x*_Fe_2_O_4,_ (D) zeta potential of Zn_1–*x*_Mg_*x*_Fe_2_O_4_ and (E) TEM images of Zn_1–*x*_Mg_*x*_Fe_2_O_4_, and the insets show the SAED patterns, respectively.

A series of Zn_1–*x*_Mg_*x*_Fe_2_O_4_ molecules were successfully synthesized as shown in Fig. 2A and the X-ray diffraction (XRD) spectra are given in Fig. 2B. Their hydrodynamic diameters measured by dynamic light scattering (DLS) were 21.96 ± 3.10 nm (MgFe_2_O_4_, polymer dispersity index (PDI): 0.02), 23.41 ± 5.04 nm (Zn_0.2_Mg_0.8_Fe_2_O_4_, PDI: 0.05), 28.52 ± 5.36 nm (Zn_0.4_Mg_0.6_Fe_2_O_4_, PDI: 0.04), 29.67 ± 6.24 nm (Zn_0.6_Mg_0.4_Fe_2_O_4_, PDI: 0.04), 40.80 ± 9.76 nm (Zn_0.8_Mg_0.2_Fe_2_O_4_, PDI: 0.06), and 48.70 ± 15.43 nm (ZnFe_2_O_4_, PDI: 0.10), as shown in Fig. 2C. The diameter of Zn_1–*x*_Mg_*x*_Fe_2_O_4_ increased with an increase in the Zn ratio. The zeta potentials of obtained Zn_1–*x*_Mg_*x*_Fe_2_O_4_ were −31.63 ± 1.31 mV (MgFe_2_O_4_), −36.5 ± 1.75 mV (Zn_0.2_Mg_0.8_Fe_2_O_4_), −29.83 ± 2.05 mV (Zn_0.4_Mg_0.6_Fe_2_O_4_), −27.93 ± 1.34 mV (Zn_0.6_Mg_0.4_Fe_2_O_4_), −30.63 ± 2.11 mV (Zn_0.8_Mg_0.2_Fe_2_O_4_), and −26.93 ± 1.57 mV (ZnFe_2_O_4_), as shown in Fig. 2D. The morphology was evaluated using a transmission electron microscope (TEM) and the selected area electron diffraction (SAED) showed that these nanoparticles had a morphology of homogeneously dispersed spheres and a single-crystal structure, as shown in Fig. 2E.

### 2.2 CAT-like, POD-like, and GSH-like properties of Zn_1–*x*_Mg_*x*_Fe_2_O_4_

#### 2.2.1 Zn_0.4_Mg_0.6_Fe_2_O_4_ exhibited excellent CAT-like activity

**Fig. 3:**
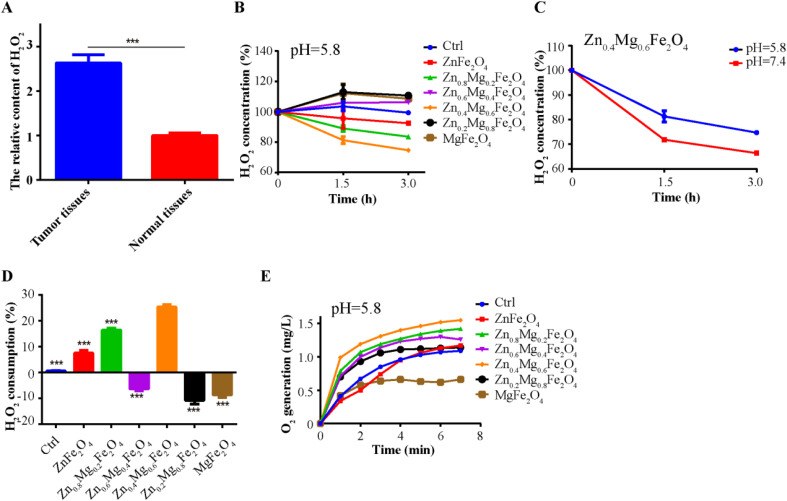
Properties of the CAT-like activity of Zn_0.4_Mg_0.6_Fe_2_O_4_. (A) The H_2_O_2_ content in OSCC tumor tissues and normal tissues. (B) H_2_O_2_ consumption continues after treatment with Zn_1–*x*_Mg_*x*_Fe_2_O_4_ in an acidic environment. (C) Continuous consumption of H_2_O_2_ after treatment with Zn_0.4_Mg_0.6_Fe_2_O_4_ in different buffers (PBS, pH = 5.8 or 7.4). (D) Comparison of CAT-like activity at 3 h. (E) Continuous generation of O_2_ after treatment with Zn_1–*x*_Mg_*x*_Fe_2_O_4_ in an acidic environment. ***: *P* < 0.001.

It is effective in reducing the tolerance to OSCC chemotherapy by increasing the content of O_2_. As shown in Fig. 3A, H_2_O_2_ was abundant in OSCC tumor tissues compared to normal tissues, which could provide the substrate for the generation of O_2_. Therefore, both the consumption of H_2_O_2_ and the generation of O_2_ have been used to assess the CAT-like activity of Zn_1–*x*_Mg_*x*_Fe_2_O_4_. In terms of consumption of H_2_O_2_, Zn_0.4_Mg_0.6_Fe_2_O_4_, Zn_0.8_Mg_0.2_Fe_2_O_4_, and ZnFe_2_O_4_ showed CAT-like activity in an acid/neutral environment (pH = 5.8/7.4) compared to the control group, while Zn_0.6_Mg_0.4_Fe_2_O_4_, Zn_0.2_Mg_0.8_Fe_2_O_4,_ and MgFe_2_O_4_ did not (Fig. 3B, C and S1†). The amount of H_2_O_2_ consumption at 3 h was used to explore the influence of the Zn/Mg ratio on the CAT-like catalytic efficiency of Zn_1–*x*_Mg_*x*_Fe_2_O_4_. As shown in Fig. 3D, Zn_0.4_Mg_0.6_Fe_2_O_4_ has decomposed the maximum amount of H_2_O_2_ than the other Zn_1–*x*_Mg_*x*_Fe_2_O_4_ nanozymes. Furthermore, as shown in Fig. 3E, Zn_0.4_Mg_0.6_Fe_2_O_4_ was also the O_2_-producing Zn_1–*x*_Mg_*x*_Fe_2_O_4_. Therefore, Zn_0.4_Mg_0.6_Fe_2_O_4_ has potential application value in correcting the tumor hypoxic microenvironment of OSCC and improving the chemotherapy effect.

#### 2.2.2 Zn_0.4_Mg_0.6_Fe_2_O_4_ exhibited excellent POD-like activity

**Fig. 4:**
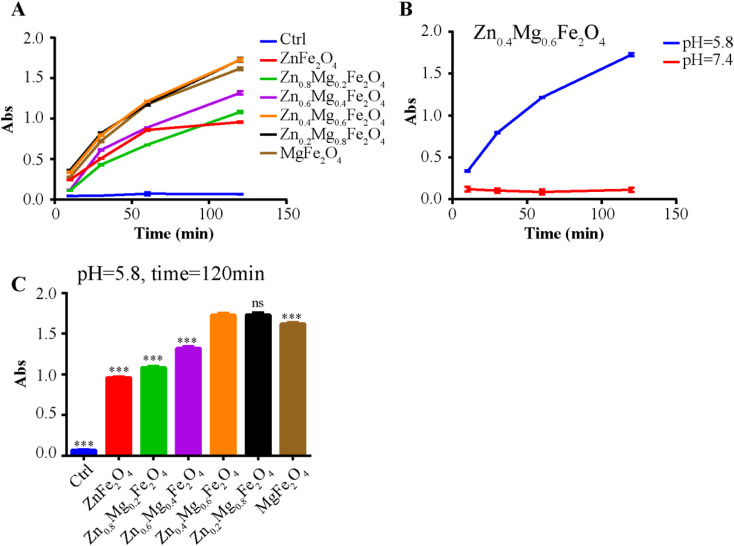
POD-like activity of Zn_0.4_Mg_0.6_Fe_2_O_4_. (A) ROS generation after treatment with Zn_1–*x*_Mg_*x*_Fe_2_O_4_ in an acidic environment. (B) Continuous ROS generation after treatment with Zn_0.4_Mg_0.6_Fe_2_O_4_ in different buffers (PBS, pH = 5.8 or 7.4). (C) Comparison of CAT-like activity at 120 min in an acidic environment.

ROS detection was performed to investigate the POD-like activity of Zn_1–*x*_Mg_*x*_Fe_2_O_4_. As shown in Fig. 4A, B and S2,† compared to the control group, all Zn_1–*x*_Mg_*x*_Fe_2_O_4_ exhibited POD-like activity in an acid environment (pH = 5.8), and no POD-like activity was observed in a neutral environment (pH = 7.4). The acid response of the POD-like activity of Zn_1–*x*_Mg_*x*_Fe_2_O_4_ ensured that the nanoenzyme would kill tumor cells only in the acidic tumor microenvironment, but would not damage normal cells. To further explore the influence of the Zn/Mg ratio on the POD-like catalytic efficiency of Zn_1–*x*_Mg_*x*_Fe_2_O_4_, the catalytic efficiency at 120 min was compared. As shown in Fig. 4C, the POD-like catalytic efficiency presented an earlier increasing and later decreasing trend with an increase of the Mg ratio; Zn_0.4_Mg_0.6_Fe_2_O_4_ and Zn_0.2_Mg_0.8_Fe_2_O_4_ had the highest POD-like catalytic efficiency.

#### 2.2.3 Zn_0.4_Mg_0.6_Fe_2_O_4_ exhibited excellent GSH-like activity in an acid environment

**Fig. 5:**
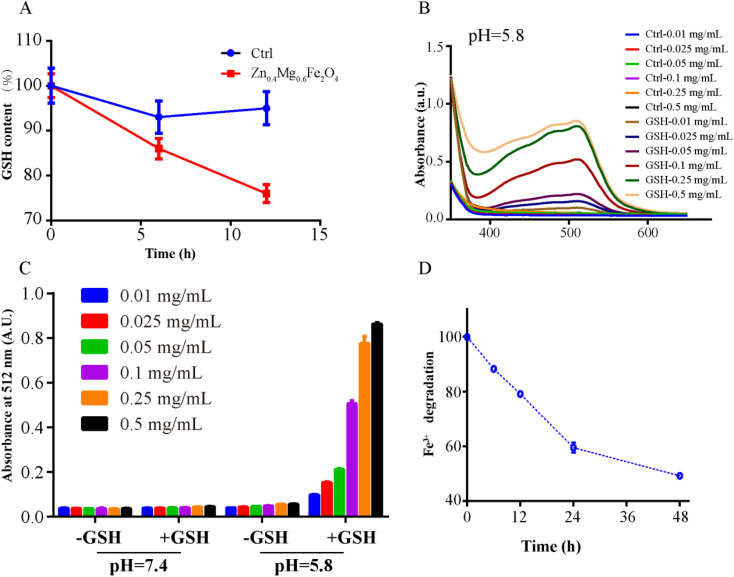
Properties of the GSH-like activity of Zn_0.4_Mg_0.6_Fe_2_O_4_. (A) GSH consumption after treatment with Zn_0.4_Mg_0.6_Fe_2_O_4_ in an acidic environment. (B) Fe^2+^ generation after treating with different concentrations of Zn_0.4_Mg_0.6_Fe_2_O_4_ in an acidic environment. (C) Fe^2+^ generation after treating with different concentrations of Zn_0.4_Mg_0.6_Fe_2_O_4_ in acidic and neutral environments. (D) Depletion of Fe^3+^ after treatment with Zn_0.4_Mg_0.6_Fe_2_O_4_.

Given the high H_2_O_2_ levels, OSCC also contained a large amount of GSH compared to normal tissues, which would increase the occurrence of chemotherapy tolerance.^16–18^ Furthermore, previous research indicated that Fe^3+^ could be reduced to Fe^2+^ by GSH and Fe^2+^ could further convert H_2_O_2_ to O_2_ or ROS.^19^ Therefore, the GSH-like activity of Zn_1–*x*_Mg_*x*_Fe_2_O_4_ was evaluated by detecting GSH consumption, Fe^2+^ generation, and Fe^3+^ depletion. As shown in Fig. 5A, GSH has been significantly consumed by Zn_0.4_Mg_0.6_Fe_2_O_4_ (14% at 6 h and 24% at 12 h) in an acidic environment. Furthermore, the generation of Fe^2+^ was also positively correlated with the concentration of Zn_0.4_Mg_0.6_Fe_2_O_4_ in an acid environment, while very little Fe^2+^ was generated in a neutral environment as shown in Fig. 5B, C and S3.† Furthermore, Fe^3+^ has also obviously been depleted after culture with GSH in acidic buffer, as shown in Fig. 5D. In summary, Zn_0.4_Mg_0.6_Fe_2_O_4_ exhibited the best enzyme-like activity among the Zn_1–*x*_Mg_*x*_Fe_2_O_4_ nanozymes which would increase sensitivity to chemotherapy.

### 2.3 Zn_0.4_Mg_0.6_Fe_2_O_4_ could shape the tumor microenvironment that inhibits the progression of OSCC

**Fig. 6:**
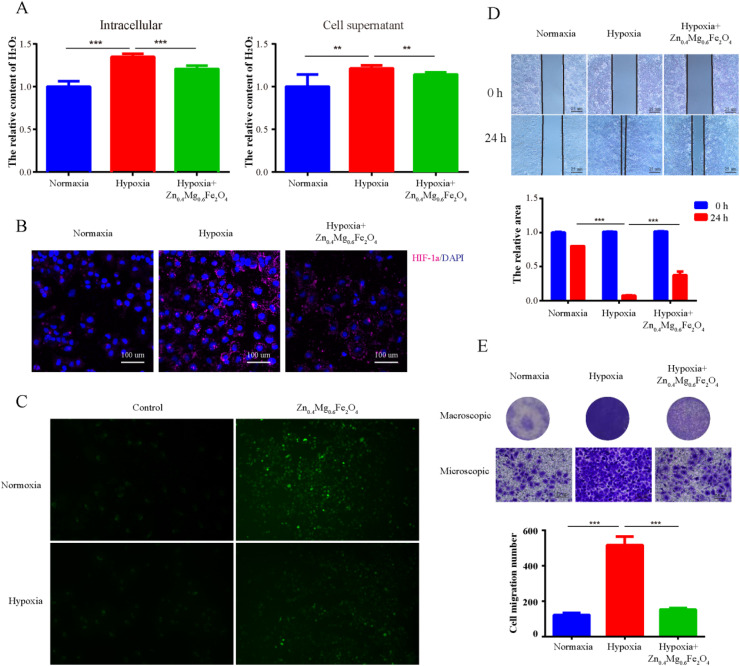
The influence of Zn_0.4_Mg_0.6_Fe_2_O_4_ on OSCC. (A) The effect of Zn_0.4_Mg_0.6_Fe_2_O_4_ on the H_2_O_2_ content, (B) on the expression of HIF-1α, (C) on ROS generation, and (D-E) on the migration ability of OSCC cells. **: *P* < 0.01, ***: *P* < 0.001.

To further validate the CAT-like activity of Zn_0.4_Mg_0.6_Fe_2_O_4_*in vitro*, the levels of H_2_O_2_ and HIF-1α were detected in OSCC tumor cells treated with 100 μg mL^−1^ Zn_0.4_Mg_0.6_Fe_2_O_4_. As shown in Fig. 6A, compared to the normoxic environment, the H_2_O_2_ content was markedly elevated in tumor cells cultured in a hypoxic environment, and was also depleted after treatment with Zn_0.4_Mg_0.6_Fe_2_O_4_. The expression of HIF-1α reflected the O_2_ content in the cells. As shown in Fig. 6B, the expression of HIF-1α in tumor cells cultured in a hypoxic environment was significantly higher than that in a normoxic environment and also decreased after treatment with Zn_0.4_Mg_0.6_Fe_2_O_4_. The ROS content was detected to validate the POD activity of Zn_0.4_Mg_0.6_Fe_2_O_4_*in vitro*. As shown in Fig. 6C, the ROS content in tumor cells was increased both in a normaxic and a hypoxic environment after treatment with Zn_0.4_Mg_0.6_Fe_2_O_4._, which further demonstrated that Zn_0.4_Mg_0.6_Fe_2_O_4_ also exhibited excellent enzyme-like activity in cells. Subsequently, a cell wound scratch assay and a transwell cell migration assay were performed to evaluate the influence of Zn_0.4_Mg_0.6_Fe_2_O_4_ on OSCC tumor cells. As shown in Fig. 6D and E, the migration rate of tumor cells increased significantly in the hypoxic environment compared to the normoxic environment, while the migration rate also decreased after treating with Zn_0.4_Mg_0.6_Fe_2_O_4_. This means that Zn_0.4_Mg_0.6_Fe_2_O_4_ may inhibit the progression of OSCC through an appropriate hypoxia environment.

### 2.4 Zn_0.4_Mg_0.6_Fe_2_O_4_ could improve the chemotherapy effect in OSCC

**Fig. 7:**
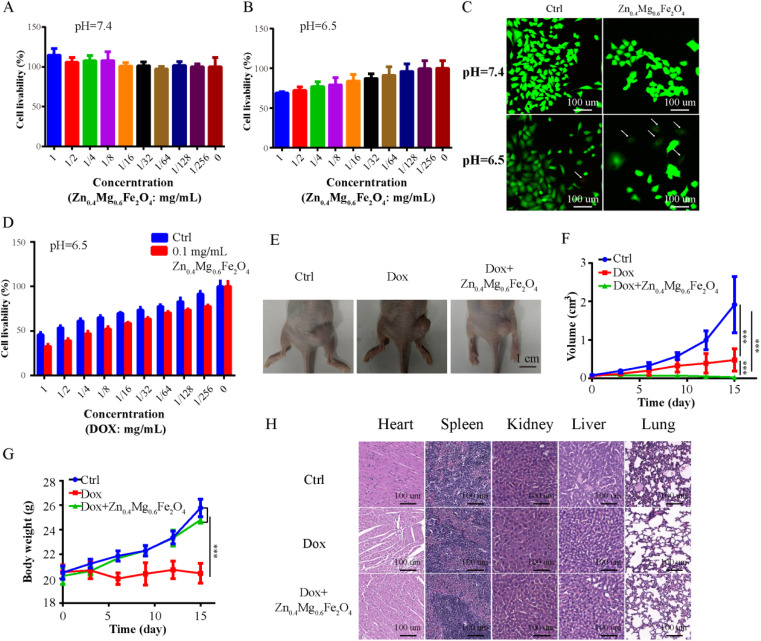
Zn_0.4_Mg_0.6_Fe_2_O_4_ could enhance the chemotherapy effect in OSCC. CCK-8 assay showing the toxicity of different concentrations of Zn_0.4_Mg_0.6_Fe_2_O_4_ in OSCC cells (A) in a neutral environment and (B) in an acidic environment. (C) Double-staining assay for live/dead cells: toxicity of 0.1 mg mL^−1^ Zn_0.4_Mg_0.6_Fe_2_O_4_ in OSCC cells in neutral and acidic environments. (D) The influence of 0.1 mg mL^−1^ Zn_0.4_Mg_0.6_Fe_2_O_4_ on the toxicity of different concentrations of DOX in OSCC cells. (E and F) Comparison of *in vivo* therapeutic efficacy between DOX and DOX + Zn_0.4_Mg_0.6_Fe_2_O_4_. (G) Comparison of body weights of DOX and DOX + Zn_0.4_Mg_0.6_Fe_2_O_4_ treatments. (H) Comparison of *in vivo* systemic toxicity of DOX and DOX + Zn_0.4_Mg_0.6_Fe_2_O_4_. Note: ***: *P* < 0.001.

In addition to surgery, chemotherapy is also an important treatment for OSCC. However, due to the tolerance to chemotherapy caused by various factors, such as hypoxia, chemotherapy outcomes are still less than satisfactory. A chemosensitizer could enhance the effect of chemotherapy. Therefore, Zn_0.4_Mg_0.6_Fe_2_O_4_ was combined with DOX (first-line chemotherapy for OSCC) to treat OSCC in this study. First, the CCK-8 assay was performed to evaluate the toxicity of Zn_0.4_Mg_0.6_Fe_2_O_4_ to OSCC cells. As shown in Fig. 7A and B, cell viability decreased as the concentration of Zn_0.4_Mg_0.6_Fe_2_O_4_ increased in an acidic environment (IC_50_-Zn_0.4_Mg_0.6_Fe_2_O_4_: 1.398 mg mL^−1^) while no toxicity was observed in a neutral environment. Furthermore, the live/dead cell-double staining assay also showed greater OSCC cell death after treatment with 0.1 mg mL^−1^ Zn_0.4_Mg_0.6_Fe_2_O_4_ in an acidic environment, as shown in Fig. 7C, which indicated that the lethal effect of Zn_0.4_Mg_0.6_Fe_2_O_4_ also had acid-response properties *in vitro*. Subsequently, we further evaluated whether Zn_0.4_Mg_0.6_Fe_2_O_4_ could enhance the chemotherapy effect of DOX on OSCC cells. As shown in Fig. 7D, compared to DOX alone (IC_50_-DOX: 0.737 mg mL^−1^), OSCC cell viability obviously decreased when combined with 0.1 mg mL^−1^ Zn_0.4_Mg_0.6_Fe_2_O_4_ (IC_50_-DOX and Zn_0.4_Mg_0.6_Fe_2_O_4_: 0.394 mg mL^−1^ and 0.1 mg mL^−1^, combination index: 0.616). Thus, Zn_0.4_Mg_0.6_Fe_2_O_4_ could efficiently enhance the chemotherapy effect of DOX on OSCC cells. Finally, a mouse xenograft OSCC tumor model was used to further validate our findings *in vivo*. As shown in Fig. 7E and F, the tumor volume of mice injected with Zn_0.4_Mg_0.6_Fe_2_O_4_ and DOX was significantly lower than that of mice injected with DOX alone, while the body weight of mice injected with Zn_0.4_Mg_0.6_Fe_2_O_4_ and DOX was higher as shown in Fig. 7G. Furthermore, cardiotoxicity was also less marked in mice injected with Zn_0.4_Mg_0.6_Fe_2_O_4_ compared to mice injected with DOX alone (Fig. 7H). Thus, Zn_0.4_Mg_0.6_Fe_2_O_4_ can not only enhance DOX chemotherapy, but can also efficiently reduce the toxicity of DOX chemotherapy *in vivo*. Therefore, Zn_0.4_Mg_0.6_Fe_2_O_4_ could serve as a chemo-sensitizer in the management of OSCC.

## 3 Experimental section

### 3.1 Synthesis of Zn_1–*x*_Mg_*x*_Fe_2_O_4_ nanozymes

The Zn_1–*x*_Mg_*x*_Fe_2_O_4_ nanozymes were prepared according to previously reported protocols with minor modifications.^20,21^ Specifically, 0.05 M Mg(NO_3_)_2_·6H_2_O/Zn(NO_3_)_2_·6H_2_O (the ratio between Mg(NO_3_)_2_·6H_2_O and Zn(NO_3_)_2_·6H_2_O was established according to the stoichiometry of Zn_1–*x*_Mg_*x*_Fe_2_O_4_), 0.1 M Fe(NO_3_)_3_ 9H_2_O and 0.15 M trisodium citrate were dissolved in 190 mL of distilled water and 10 mL of 1 M NaOH solution was then slowly added to the solution. The reactants were then placed in a 500-mL capacity container in a Teflon-lined autoclave. The autoclave was maintained at 160 °C for 6 h to prepare MgFe_2_O_4_, 180 °C for 8 h to prepare Zn_0.2_Mg_0.8_Fe_2_O_4_ and Zn_0.4_Mg_0.6_Fe_2_O_4_, and 180 °C for 6 h to prepare Zn_0.6_Mg_0.4_Fe_2_O_4_, Zn_0.8_Mg_0.2_Fe_2_O_4_, and ZnFe_2_O_4_.

### 3.2 Characterization of Zn_1–*x*_Mg_*x*_Fe_2_O_4_ nanozymes

The structural characterization and phase identifications of the sample are done with an X-ray diffractometer (Bruker AKS D8 Advance). The hydrodynamic diameter and zeta potential were measured using a dynamic light scattering instrument (DLS) (ZetaSizer Nano-ZS90; Malvern Instrument) in zeta potential analysis mode. The size and morphology of the obtained Zn_1–*x*_Mg_*x*_Fe_2_O_4_ nanozymes were observed by transmission electron microscopy (TEM; JEOL).

### 3.3 H_2_O_2_ consumption

The H_2_O_2_ consumption was measured using the colorimetric method of titanium sulfate (Ti(SO_4_)_2_) according to a previous protocol with minor modifications.^22^ Specifically, solution A: 2 mg of Zn_1–*x*_Mg_*x*_Fe_2_O_4_ was incubated with 5 mL of H_2_O_2_ (1 mM) in different buffers (pH 7.4 or pH 5.8) and stirred at 37 °C. Solution B: 665 μL of Ti(SO_4_)_2_ and 4.165 mL of H_2_SO_4_ were mixed with 25 mL of water. Then 100 μL of solution A was added to 200 μL of solution B at 1.5 h and 3 h. Ten minutes after addition, Zn_1–*x*_Mg_*x*_Fe_2_O_4_ was separated by centrifugation. UV-vis spectra were recorded to measure the remaining H_2_O_2_ by measuring the absorbance at 405 nm.

### 3.4 O_2_ generation

The generation of O_2_ was measured according to a method reported in the previous literature^22^ with minor modifications. In summary, 4 mg of Zn_1–*x*_Mg_*x*_Fe_2_O_4_ was incubated with 20 mL of PBS (pH = 5.8) containing 0.5 mM H_2_O_2_ at 37 °C. The dissolved O_2_ concentration was monitored with an oxygen meter (HI9146, HANNA instruments, Korea) in real time at 60, 120, 180, 240, 300, 360, and 420 min.

### 3.5 POD-like activity detection

The TMB assay was used to detect the POD-like activity of Zn_1–*x*_Mg_*x*_Fe_2_O_4_. In brief, 20 μg of Zn_1–*x*_Mg_*x*_Fe_2_O_4_ was incubated in different buffers (pH 5.8 or pH 7.4) containing 1 mM TMB and 50 mM H_2_O_2_. POD-like activity was measured by detecting the absorbance at 625 nm at 30, 60, and 120 min.

### 3.6 GSH-like properties

GSH consumption, Fe^2+^ generation and Fe^3+^ depletion were investigated to detect GSH-like activity of Zn_0.4_Mg_0.6_Fe_2_O_4_.^19,22^ To investigate the consumption of GSH, 475 μL of Zn_0.4_Mg_0.6_Fe_2_O_4_ (10 μg mL^−1^) was incubated with 25 μL of GSH (4 mM) at 37 °C for 6 and 12 h. Zn_0.4_Mg_0.6_Fe_2_O_4_ was separated by centrifugation and the supernatant was collected for GSH measurement using the GSH kit (Beyotime Biotechnology). Finally, 10 μL of the supernatant was added to 100 μL of the reaction mixture from the GSH kit for 25 min and the concentration of GSH was measured by UV-vis spectroscopy at 410 nm. To investigate Fe^2+^ generation, different concentrations of Zn_0.4_Mg_0.6_Fe_2_O_4_ (0.01, 0.025, 0.05, 0.1, 0.25, and 0.5 mg mL^−1^) were incubated with/without 4 mM GSH in different buffers (pH 5.8 or pH 7.4) at 25 °C for 1 h, then the solutions were centrifuged (15 000 rpm, 20 min) and the supernatant was collected. A 100-μL volume of saturated 1, 10-phenanthroline solution was added to the supernatant. The absorbance at 512 nm was subsequently monitored. To investigate Fe^3+^ depletion, 10 μg mL^−1^ Zn_0.4_Mg_0.6_Fe_2_O_4_ was cultured with 4 mM GSH in acidic buffer (pH = 5.8) at 25 °C for 12, 24, 36, and 48 h. Then the Fe^2+^ generation was quantitatively analyzed according to the standard Fe^2+^ concentration absorbance curve, and the Fe^3+^ depletion was equal to the Fe^3+^ generation.

### 3.7 Cell culture

The OSCC cell line SCC9 was obtained from Fudan University (Shanghai, China); SCC9 cells were cultured in DMEM medium (Gibco) supplemented with 1% FBS (Sigma) in a humidified incubator at 37 °C with 5% CO_2_. All the cell lines tested negative for mycoplasma contamination.

### 3.8 The detection of H_2_O_2_ concentration in tissues and cells

The H_2_O_2_ concentration in tissues and cells was measured using an H_2_O_2_ assay kit (S0038, Beyotime) according to the manufacturer's protocol. Specifically, for the detection of H_2_O_2_ in tumor tissues and normal tissues acquired from OSCC patients, tissue samples were first homogenized with lysis buffer supplied by the H_2_O_2_ assay kit (the ratio was 100 μL of lysate to 5 mg of tissues), and then centrifuged at 12 000 rpm for 5 min. All operations were performed on ice. The supernatant was collected for subsequent measurement of H_2_O_2_.

This study was conducted in accordance with the Declaration of Helsinki and was approved by the medical ethics committee of the Institute Affiliated Stomatology Hospital, Medical School of Nanjing University. Written informed consent was obtained from all patients. For extracellular and intracellular H_2_O_2_ measurement in tumor cells, SCC9 cells were first incubated in fresh culture medium in a normoxic environment (21% O_2_), in fresh culture medium in a hypoxic environment (1% O_2_), and in fresh culture medium supplemented with 100 μL of Zn_0.4_Mg_0.6_Fe_2_O_4_ in a hypoxic environment for 24 h. The culture medium was then harvested to determine the extracellular H_2_O_2_ concentration. Subsequently, cells were lysed in 100 μL of lysis buffer and supernatants, collected by centrifuging at 12 000×*g* for 10 min, and were used to determine the intracellular H_2_O_2_ concentration. The H_2_O_2_ concentration detection step was as follows: 50 μL of sample solution was incubated with 100 μL of reaction solution at room temperature for 30 min and then absorption at 560 nm was measured.

### 3.9 Detection of HIF-1α expression

HIF-1α protein expression was detected by the immunofluorescence assay. Specifically, 1 × 10^3^ cells (SCC9) were seeded in glass bottom culture plates. After 24 h of culture, cells were cultured in a normoxic environment, hypoxic environment, and hypoxic environment supplemented with 100 μg mL^−1^ Zn_0.4_Mg_0.6_Fe_2_O_4_ for an additional 24 h. The cells were then fixed with 4% paraformaldehyde for 20 min and blocked with 5% BSA for 30 min at room temperature. Cells were incubated with the primary antibody HIF-1α (Cat no.: 66730-1-Ig, Proteintech) overnight at 4 °C. Dylight-conjugated anti-mouse IgG (647-conjugated anti-mouse IgG, Abcam, USA) was used as the secondary antibody. The nucleus was stained with DAPI (Beyotime, China) and observed under a confocal scanning system (Ti, NIKON).

### 3.10 Intracellular ROS detection

Intracellular ROS expression was detected using a reactive oxygen species assay kit (Beyotime, China). In summary, 1 × 10^3^ cells (SCC9) were seeded in glass bottom culture plates. After 24 h of culture, cells were placed in a normoxic environment, a normoxic environment supplemented with 100 μg mL^−1^ Zn_0.4_Mg_0.6_Fe_2_O_4_, a hypoxic environment, and in a hypoxic environment supplemented with 100 μg mL^−1^ Zn_0.4_Mg_0.6_Fe_2_O_4_ for an additional 24 h. The DCFH-DA probe (10 μM) was then added to the cells and incubated for another 30 min at 37 °C. Cells were washed three times with PBS and monitored using a fluorescence microscope (Ti2, NIKON) with 488 nm excitation.

### 3.11 Cell wound healing assay and transwell cell migration assay

The migration ability of OSCC cells was determined by the cell wound healing assay and the transwell cell migration assay. For the cell wound healing assay, Ibidi Culture-Insert 2 wells were placed in 6-well plates, and then SCC9 cells were seeded for 24 hours before forming a 500-μm cell scratch on the cell layer surface. Next, cells were cultured with a fresh medium in a normoxic environment, a hypoxic environment, and a hypoxic environment supplemented with 100 μg mL^−1^ Zn_0.4_Mg_0.6_Fe_2_O_4_. Phase-contrast images were acquired at the time of the scratch and 24 h later. For the transwell cell migration assay, 100 μL of SCC9 was seeded in the upper chamber with 10% FBS in the lower chamber in a normoxic environment, a hypoxic environment, and a hypoxic environment supplemented with 100 μg mL^−1^ Zn_0.4_Mg_0.6_Fe_2_O_4_. Twenty-four hours later, the cells at the bottom of the filter were stained with crystal violet staining solution (Beyotime, China), and the cell numbers were counted in five fields by microscopy.

### 3.12 Cell proliferation assay

The proliferation ability of OSCC cells was determined using a CCK-8 cell proliferation assay kit (Beyotime). Briefly, 1 × 10^3^ SCC9 cells were placed in a 96-well plate and cultured for 24 h. The culture medium was then replaced with fresh medium (pH 6.5 or pH 7.4) supplemented with a different concentration of Zn_0.4_Mg_0.6_Fe_2_O_4_ (0–1 mg mL^−1^) or different concentrations of DOX (0–1 mg mL^−1^). After 1 day of incubation, the culture medium was aspirated and treated cells were washed with PBS, before the addition of 10 μL of CCK8 solution to each well and incubated for an additional 2 hours at 37 °C. The absorbance of each well at 450 nm was then measured using an IMark enzyme mark instrument (Bio-Rad Inc., USA). The cell proliferation ability was calculated on the basis of the absorbance data.

### 3.13 Live/dead cell double staining assay

A Calcein-AM/PI double staining kit (Cat no.: 40747ES76, Yeasen, China) was used to detect live/dead cells. Briefly, 1 × 10^3^ cells (SCC9) were seeded in glass bottom culture plates. After 24 h of culture, cells were cultured in a neutral environment, in a neutral environment supplemented with 100 μg mL^−1^ Zn_0.4_Mg_0.6_Fe_2_O_4_, in an acidic environment, and in an acidic environment supplemented with 100 μg mL^−1^ Zn_0.4_Mg_0.6_Fe_2_O_4_ for an additional 24 h. The cells were then washed with 1× assay buffer three times and incubated with 100 μL of detection buffer (5 μL of 2 mM Calcein-AM solution and 15 μL of 1.5 mM PI solution in 5 mL of assay buffer) for 15 min at 3 °C and observed under a confocal scanning system.

### 3.14 Animal model

BALB/c athymic nude mice were provided by the Comparative Medical Center of YangZhou University. All mice were housed under SPF conditions in which food, water, bedding, and cages were irradiated prior to use. All animal procedures were performed according to Nanjing University Laboratory Animal Care and Use Guidelines and the experiments were approved by the Nanjing University Animal Ethics Committee. To investigate antitumor efficacy, mice with subcutaneous OSCC xenografts were randomly divided into three groups (*n* = 5 for each group) when the tumor reached approximately 0.05 cm^3^: (1) PBS group (intravenous injection of 100 μL of PBS), (2) DOX group (intravenous injection of 100 μL of PBS containing 0.25 mg kg^−1^ DOX) and (3) DOX + Zn_0.4_Mg_0.6_Fe_2_O_4_ group (intravenous injection of 100 μL of PBS containing 0.25 mg kg^−1^ DOX and 0.5 mg kg^−1^ Zn_0.4_Mg_0.6_Fe_2_O_4_). The drugs were administered every 3 days for 5 times and the tumor volumes/body weight were recorded simultaneously. Tumor volumes were calculated using the following formula: *V* = 0.5*ab*^2^ (*a*: the longest diameter and *b*: the shortest diameter). To examine histological changes in the organs of the experimental mice, including the heart, liver, spleen, lung and kidney, 15 days after drug administration, organs were collected from mice and samples were embedded in paraffin. Subsequently, the paraffin-embedded tissue sections were stained with H&E and observed using a light microscope.

### 3.15 Statistical analysis

Statistical analysis was performed using SPSS statistical software (version 23.0; IBM) and GraphPad Prism (Version 6.0, GraphPad Software, La Jolla, CA, USA). Student's two-sided *t*-test was used to evaluate statistical significance differences between two groups and one-way ANOVA was used to evaluate statistical significance differences between three or more groups. MANOVA was used to analyze the repeated measured data. A *P*-value ≤0.05 was considered statistically significant.

## 4 Conclusions

Zn_0.4_Mg_0.6_Fe_2_O_4_ possessed optimal enzyme-like activity including CAT-like, POD-like, and GSH-like activity across the series of Zn_1–*x*_Mg_*x*_Fe_2_O_4_ nanoenzymes in an acidic environment. Furthermore, Zn_0.4_Mg_0.6_Fe_2_O_4_ could effectively improve the efficacy of DOX chemotherapy in the treatment of OSCC and reduce cardiotoxicity. Zn_0.4_Mg_0.6_Fe_2_O_4_ could serve as a promising alternative chemosensitizer in the management of OSCC.

## Author contributions

Liang Chen: conceptualization, data curation, and writing - original draft. Qingmei Kong: validation and formal analysis. Mingxing Tian: formal analysis and methodology. Qian Zhang: validation. Chengwan Xia: formal analysis and review & editing. Chao Deng: methodology, project administration, review & editing, funding acquisition, and supervision.

## Conflicts of interest

The authors declare that they have no known competing financial interests or personal relationships that could have appeared to influence the work reported in this paper.

## Supplementary Material

NA-005-D2NA00750A-s001
