# Supplementary material for: Zn0.4Mg0.6Fe2O4 nanoenzyme: a novel chemo-sensitizer for the chemotherapy treatment of oral squamous cell carcinoma
Source: Nanoscale Adv. 2022 Dec 28;5(3):851–60. doi: 10.1039/d2na00750a (PMC9890649; doi:10.1039/d2na00750a)
Supplement: NA-005-D2NA00750A-s001 [file NA-005-D2NA00750A-s001.pdf]

Electronic Supplementary Material (ESI) for Nanoscale Advances.
This journal is © The Royal Society of Chemistry 2022

$Zn_{0.4}Mg_{0.6}Fe_2O_4$ nanoenzyme: a novel chemo-sensitizer for the chemotherapy treatment of Oral Squamous Cell Carcinoma

Liang Chen,‡[a] Qingmei Kong,‡[b] , Mingxing Tian[a], Qian Zhang,[b] Chengwan Xia,*[b] and Chao Deng*[c]

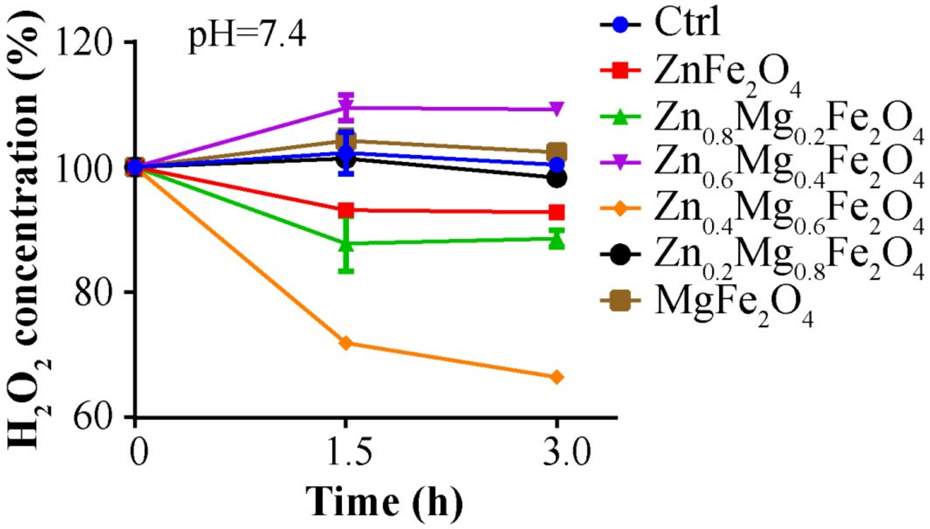

Fig.S1 Continues H_2O_2 consumption after treating with $Zn_{1-x}Mg_xFe_2O_4$ under neutral environment.

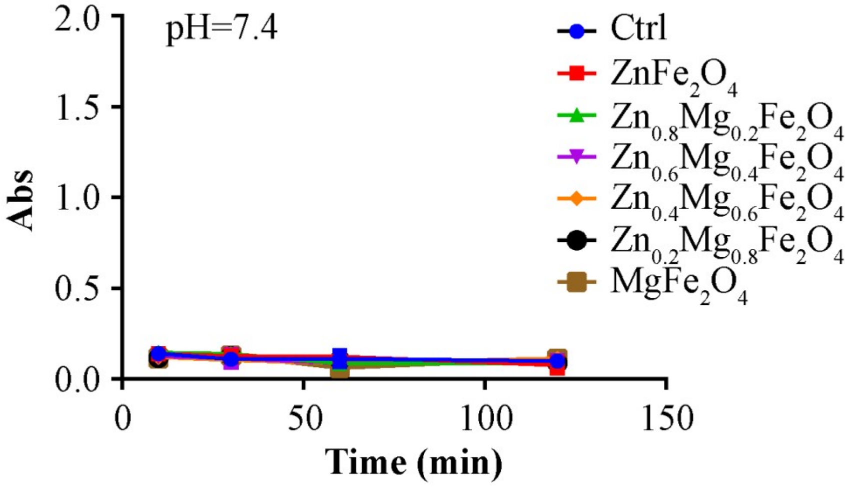

Fig.S2 Continues ROS generation after treating with $Zn_{1-x}Mg_xFe_2O_4$ under neutral environment.

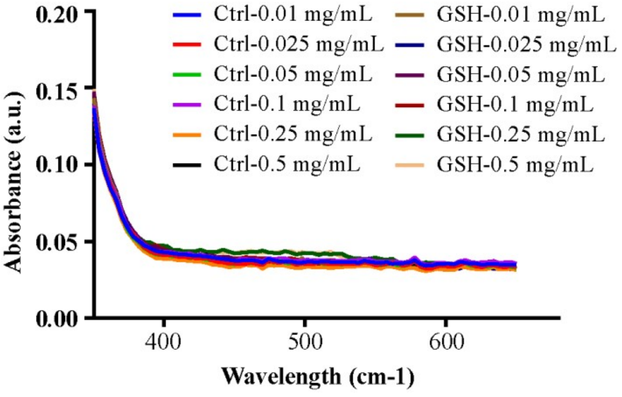

Fig.S3 Fe^{2+} generation after treating with different concentration of $Zn_{0.4}Mg_{0.6}Fe_2O_4$ under neutral environment.
